# An Open-Circuit Fault Diagnosis Method for Three-Level Neutral Point Clamped Inverters Based on Multi-Scale Shuffled Convolutional Neural Network

**DOI:** 10.3390/s24061745

**Published:** 2024-03-07

**Authors:** Yan Yan, Jiaqi Wu, Yanfei Cao, Bo Liu, Chen Li, Tingna Shi

**Affiliations:** College of Electrical Engineering, Zhejiang University, Hangzhou 310027, China; yan_yan@zju.edu.cn (Y.Y.); 22110148@zju.edu.cn (J.W.); caoyanfei@zju.edu.cn (Y.C.); liub21@zju.edu.cn (B.L.); lichen_hz@zju.edu.cn (C.L.)

**Keywords:** NPC inverter, open-circuit fault, convolutional neural network, fault diagnosis

## Abstract

This study constructs a power switching device open-circuit fault diagnosis model for a three-level neutral point clamped inverter based on the multi-scale shuffled convolutional neural network (MSSCNN) and extracts and classifies the fault information contained in the output current of inverters. The model employs depthwise separable convolution and channel shuffle techniques to improve diagnostic accuracy and reduce model complexity. The experimental results show that the new model has lower model complexity, better noise resistance and higher average diagnostic accuracy compared with fault diagnosis models based on CNN, ResNet, ShuffleNet V2 and Mobilenet V3 networks.

## 1. Introduction

The three-level neutral point clamped (NPC) inverter has been widely recognized and applied in the industry due to its high withstand voltage level, low output harmonic content and flexible control methods [1,2]. Power switching devices are one of the most vulnerable parts of power converter systems [3]. Once a fault occurs, it will cause the entire power converter system to malfunction, potentially damaging the system and threatening personal safety. Given that the three-level NPC inverter employs a greater number of power switching devices compared to the two-level inverter, the probability of failures is higher. Therefore, timely and accurate diagnosis and detection of power switching device faults in the three-level NPC inverter are important for enhancing the reliability and safety of the system operation.

Power switching device faults are primarily categorized into open-circuit and short-circuit faults [4]. In the event of a short-circuit fault, the instantaneous overcurrent can cause severe damage to the inverter. In practical applications, rapid protection is typically achieved using hardware such as fuses. On the other hand, open-circuit faults generally do not immediately lead to system breakdown. However, the output current distortion introduced by open-circuit faults, if not promptly diagnosed, can also result in damage to the inverter or the load. As a result, the focus of most research is predominantly on the diagnosis of open-circuit faults. The diagnostic methods for open-circuit faults can mainly be divided into three categories: model-based, signal-based and artificial intelligence (AI)-based analysis.

Model-based fault diagnosis methods use the mathematical model of the inverter to estimate physical quantities such as voltage or current. By comparing the residual between the estimated values and the actual signals, it can determine whether a fault has occurred and locate the faulty power devices. In [5], a novel adaptive sliding mode observer is proposed to estimate three-phase currents. To ensure the sensitivity and robustness of the fault detection method, an adaptive threshold strategy is designed. In [6], the DC-side voltage is estimated by utilizing PWM signals and current measurements. The estimated value is then compared with the measured value, and fault diagnosis is achieved through voltage residuals. In [7], a dynamic model of a single-phase NPC converter is established, and fault diagnosis is implemented by calculating the rate of change in current residuals based on this model. In [8], fault detection is achieved by using a method based on model calculations of instantaneous output voltage errors. The aforementioned methods require the establishment of accurate mathematical models, exhibit a strong dependency on physical parameters and demand high sampling frequencies.

The signal analysis-based fault diagnosis method entails the analysis of diverse inverter signals using signal processing techniques, followed by the extraction of key fault characteristics. The diagnosis is then achieved by establishing the correspondence between the extracted fault features and the types of faults. In [9], fault detection and localization are implemented by analyzing the changes in the DC bus capacitor voltage and load current after an open-circuit fault in the inverter. In [10], fault detection and the localization of the faulty bridge arm are carried out by calculating the amplitude and phase angle of the current after Park transformation. This is then combined with the average value of the normalized current to locate the faulty device. In [11], current vector radius variation and switching state injection are used to achieve precise fault device localization. Signal-based methods exhibit a strong dependence on the load connected to the inverter output, and their diagnostic effectiveness is easily affected by environmental noise and system disturbances.

AI-based fault diagnosis methods involve hierarchical processing of fault data, gradually extracting and categorizing relevant data features. Intelligent judgments and decisions are then made by establishing a mapping relationship between input data and fault categories. The work in [12] utilizes wavelet packet energy spectrum entropy to extract the characteristics of the midpoint voltage signal in the bridge arm and employs kernel principal component analysis for a dimensionality reduction in the feature vector. Subsequently, fault classification is performed using a wavelet neural network. In [13], the location of the faulty submodule in a modular multilevel converter (MMC) is determined by extracting time-domain features such as variance and mean from the submodule capacitor voltage data and constructing a random forest classifier. The methods mentioned above require complex processing of raw data, increasing the implementation difficulty. Convolutional neural networks (CNNs) have garnered attention for their powerful feature extraction capabilities [14]. In [15], a diagnostic method is proposed for rotating machinery, which involves the direct classification of continuous wavelet transform scaleograms (CWTS) using CNN. In [16], the combination of a CNN with a discrete wavelet transform enabled the identification of open-circuit faults in inverters. However, this method necessitates the generation of current vector trajectory maps, thereby increasing the complexity of data processing. The work in [17] applies a CNN based on the inception structure for diagnosing open-circuit faults in PWM converters. However, the diagnostic speed is influenced significantly due to the network’s extensive parallel structure. In [18], a one-dimensional CNN with an improved stochastic gradient optimization method is introduced for extracting and classifying inverter fault features. In [19], a multimodal deep residual filter network (DRFN) is proposed, achieving a 99.18% accuracy rate in identifying open-circuit faults in T-type three-level inverters. However, this approach requires the collection of voltage and current data and involves a complex model with high computational demands.

In response to the challenges posed by existing AI-based fault diagnosis methods, such as high model complexity and cumbersome data processing, this paper proposes a diagnostic approach for open-circuit faults in power switching devices of three-level NPC inverters. The method is based on a multi-scale shuffled convolutional neural network (MSSCNN). This approach efficiently extracts features using depthwise separable convolution and channel shuffle techniques. It combines convolution kernels of different sizes to enhance the algorithm’s diagnostic effectiveness. Current data, preprocessed for simplicity, are compiled into a dataset for model training and testing. Subsequently, the proposed fault diagnosis method is tested on this dataset alongside the existing CNN [20], ResNet [21], ShuffleNet V2 [22] and MobileNet V3 [23]. The effectiveness and application results of the proposed diagnostic method are validated across three dimensions: model complexity, fault recognition accuracy and noise resistance.

## 2. Working Principle and Analysis of Fault Characteristics of Three-Level NPC Inverter

### 2.1. Working Principle

**Figure 1:**
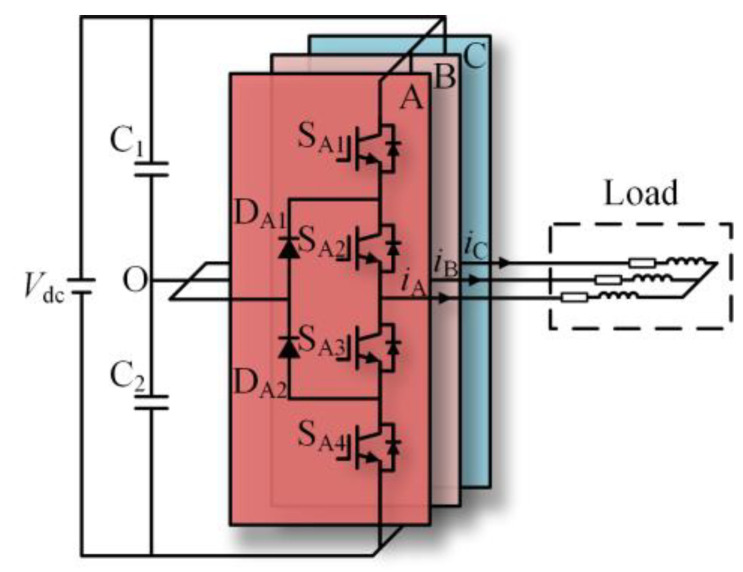
Topology of the three-level NPC inverter.

The topology of the three-level NPC inverter is shown in Figure 1. Each phase has four switching devices, S*_x_*_1_, S*_x_*_2_, S*_x_*_3_, S*_x_*_4_ (*x* = A, B, C), and two clamping diodes, D*_x_*_1_ and D*_x_*_2_. Table 1 shows the three voltage levels that the three-level NPC inverter can output.

The desired output voltages of the three-level NPC inverter can be expressed as

(1) $$\left\{\begin{array}{l} v_{A}^{*}=V_{m}^{*}\cos\left(\omega^{*}t+\varphi_{0}\right)\\ v_{B}^{*}=V_{m}^{*}\cos\left(\omega^{*}t+\varphi_{0}-2\pi /3\right)\\ v_{C}^{*}=V_{m}^{*}\cos\left(\omega^{*}t+\varphi_{0}+2\pi /3\right) \end{array}\right.$$

where *V*_m_* is the desired amplitude of the three-phase sinusoidal reference voltage; ω* is the angular frequency; and *φ*_0_ is the initial phase of the A-phase voltage.

The modulation waveform expression under the space vector modulation strategy can be expressed as follows

(2) $$\left\{\begin{array}{l} {v^{\prime }}_{A}=v_{A}^{*}+v_{Z}=m\frac{2V_{\mathrm{dc}}}{\sqrt{3}}\cos\left(\omega^{*}t+\varphi_{0}\right)+v_{Z}\\ {v^{\prime }}_{B}=v_{B}^{*}+v_{Z}=m\frac{2V_{\mathrm{dc}}}{\sqrt{3}}\cos\left(\omega^{*}t+\varphi_{0}-2\pi /3\right)+v_{Z}\\ {v^{\prime }}_{C}=v_{C}^{*}+v_{Z}=m\frac{2V_{\mathrm{dc}}}{\sqrt{3}}\cos\left(\omega^{*}t+\varphi_{0}+2\pi /3\right)+v_{Z} \end{array}\right.$$

(3) $$m=\sqrt{3}V_{m}^{*}/2V_{\mathrm{dc}}$$

(4) $$v_{Z}=-\frac{v_{\max}+v_{\min}}{2}$$

where *m* is the modulation index; *v*_z_ is the zero-sequence voltage, obtained by calculating the maximum value *v*_max_ and minimum value *v*_min_ of the three-phase sinusoidal reference voltages *V*_A_^*^, *V*_B_^*^ and *V*_C_^*^ at any given time.

**Figure 2:**
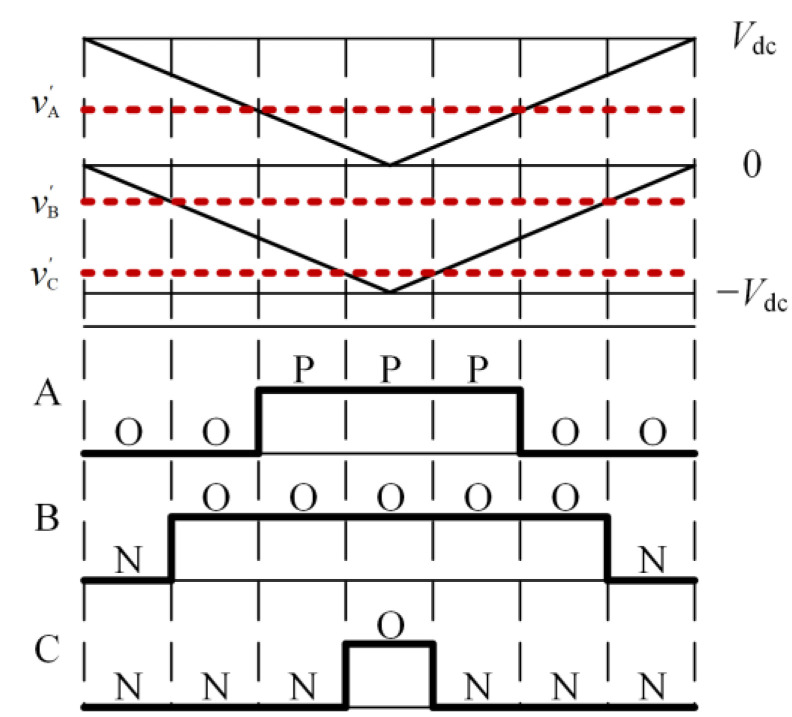
Switch sequence generation.

By comparing the modulation wave obtained from (2) with two stacked triangular carriers in the same direction and with equal amplitude, the switching sequence of each bridge arm can be determined, as shown in Figure 2.

### 2.2. Analysis of Fault Characteristics

When an open-circuit fault occurs in the power switching device, the current flow path will change, thus affecting the output voltage level of the inverter. The direction of current flow from the inverter to the load is defined as the positive direction of current (*i_x_* > 0).

**Figure 3:**
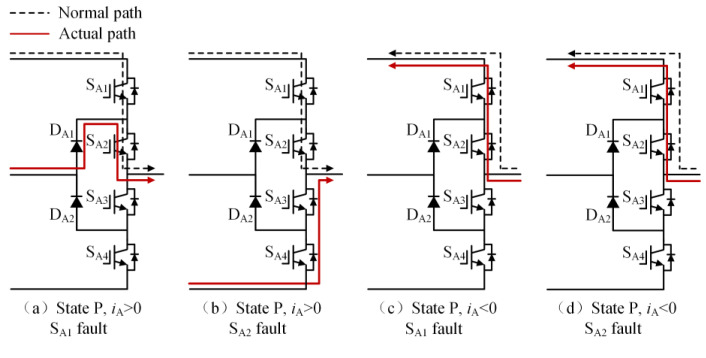
The current path when S_A1_ or S_A2_ experiences an open-circuit fault in the P state.

Taking the open-circuit fault of S_A1_ in the P state as an example, when the current is in the positive direction, the current will flow from the clamp diode D_A1_ and S_A2_ to the load. The actual state will change from a P to O state, and the output voltage will drop from *V*_dc_/2 to 0, as shown in Figure 3a; when the S_A2_ has an open-circuit fault in the P state, if the current is in the positive direction, the current will flow from the anti-parallel diodes of S_A3_ and S_A4_ to the load, the actual state will change from P to N state and the output voltage will drop from *V*_dc_/2 to −*V*_dc_/2, as shown in Figure 3b. If the current is negative, neither S_A1_ nor S_A2_ experiencing an open-circuit fault will impact the output of the inverter, as shown in Figure 3c,d. Table 2 summarizes the changes in the inverter’s output voltage before and after a single-device open-circuit fault. Taking phase A as an example, Table 3 presents the simulation waveforms of the inverter’s three-phase output current when four switching devices experience open-circuit faults successively. Throughout the simulation process, the modulation index is 0.6, the output fundamental frequency is 50 Hz and the switching frequency is 2 kHz. The load comprises a resistive-inductive configuration, with 22 Ω resistance and 4 mH inductance. It can be observed that the current waveform exhibits varying degrees of distortion, and when connected to the motor load, this distortion will cause significant torque/speed fluctuations.

## 3. Diagnosis Method for Open-Circuit Faults in Three-Level NPC Inverter Based on MSSCNN

The fault diagnosis method proposed in this paper utilizes high-precision current sensors to collect the three-phase output current data of the inverter and combines them with the proposed neural network fault diagnosis model to achieve the identification and fault localization of twelve types of open-circuit faults (as shown in Table 4) occurring in the inverter power switching devices.

**Figure 4:**
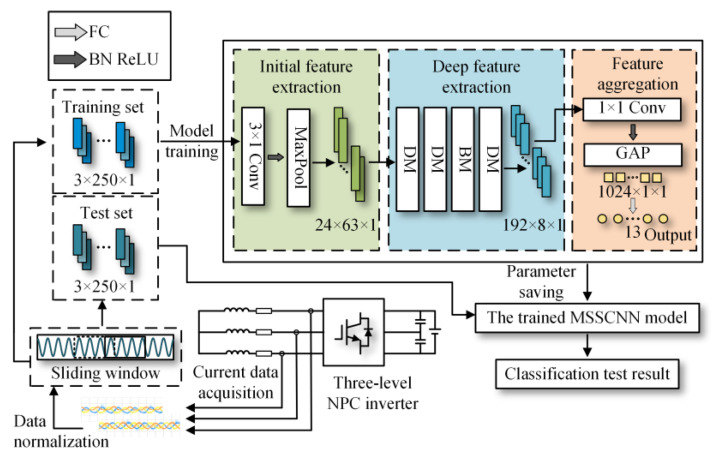
The development process of the open-circuit fault diagnosis method for three-level NPC inverter devices based on MSSCNN.

The fault diagnosis method framework is shown in Figure 4. The development process of the method includes the following:

1. Collection of three-phase current data under different operating conditions, both with open-circuit faults and fault-free states.
2. Normalization of the collected current data, and selection of a portion of the data from different operating conditions as the training set, while the remaining data are used as the test set.
3. Building the MSSCNN model on the PyCharm platform, which consists of three main parts: initial feature extraction, deep feature extraction and feature aggregation and output. Taking into account the effectiveness of feature extraction, classification and computational complexity, the number of output channels for these three parts is set to 24, 192 and 1024, respectively. The model initially extracts features from the input current data using ordinary convolution, then further extracts high-dimensional fault information features using the MSSCNN basic module (BM) and downsampling module (DM). Finally, it aggregates the extracted information and outputs the diagnostic result.
4. The training set is used to train the model, and the diagnostic effect of the model is verified through the test set.

### 3.1. Data Preprocessing

To improve the accuracy of the diagnostic network, it is necessary to preprocess the three-phase current data output by the inverter.

#### 3.1.1. Data Normalization

The collected current data from any phase are represented as a one-dimensional sequence using the following equation

(5) $$X^{k}=\left(X_{1}^{k},X_{2}^{k},\ldots ,X_{i}^{k},\ldots X_{L_{data}}^{k}\right)$$

where *k*∈{1, 2, 3} is the channel number, with channels 1, 2 and 3 corresponding to phases A, B and C, respectively; $X_{i}^{k}$ represents the *i*-th data in the *k*-th channel; *L*_data_ represents the length of the sequence data.

To reduce the impact of current amplitude variations on diagnostic efficiency and accuracy, linear normalization is performed on the collected raw data in this paper, with the expression as follows

(6) $$X_{i}^{k*}=\frac{X_{i}^{k}-X_{\min}^{k}}{X_{\max}^{k}-X_{\min}^{k}}$$

where $X_{i}^{k*}$ represents the normalized data; $X_{\max}^{k}$ and $X_{\min}^{k}$ represent the maximum and minimum values in the data of the *k*-th channel, respectively.

#### 3.1.2. Dataset Production

In the normalized data sequence $X^{k*}$, resampling is performed at every *n*_interval_ data point, with the expression as follows

(7) $$n_{\mathrm{interval}}=\frac{f_{s}}{f_{res}}$$

where *f*_s_ represents the original sampling frequency of the data sequence; *n*_interval_ represents the interval count; *f*_res_ is the resampling frequency, and its value should satisfy the condition that the newly generated sequence *L*_res_ contains *n* normalized data points within one fundamental cycle, with the expression as follows

(8) $$f_{res}=fn$$

where *f* represents the fundamental frequency; *n* represents the length of data within one fundamental cycle.

**Figure 5:**
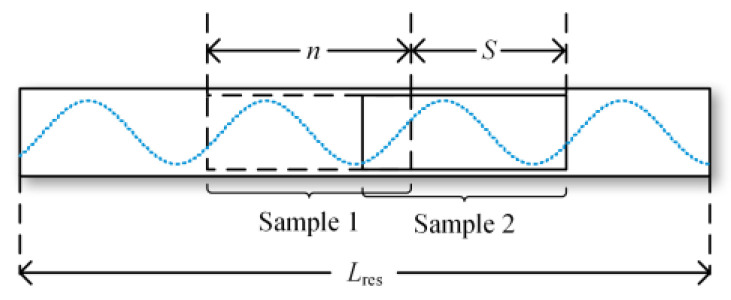
Sliding window.

After completing the resampling of the normalized data, different data segments are obtained through a sliding window approach, as shown in Figure 5. Data are cut using a sliding window with a length of *n,* and, thus, each generated sample has a length of *n*. The sliding window continuously moves over the resampled data sequence to generate different samples, with each slide set at a distance of *S*. In the method proposed in this paper, considering the computational load of the GPU, *n* is set to 250, *S* to 10 and the value of *n*_interval_ is determined based on the fundamental frequency.

### 3.2. MSSCNN Fault Diagnosis Model

**Figure 6:**
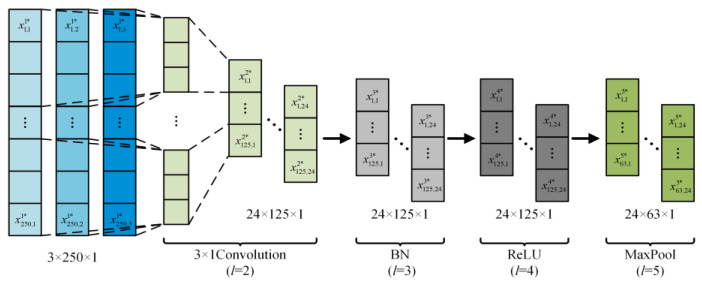
Feature preliminary extraction structure.

As shown in Figure 6, the MSSCNN fault diagnosis model proposed in this paper initially uses 3 × 1 convolution to extract shallow features from the sample data. With a stride of 2, the data length in the convolution layer is halved to a value of 125. The output data size of this layer is 24 (number of channels) × 125 (data length) × 1 (dimension). Furthermore, batch normalization (BN) is applied to normalize these shallow features [24]. The BN layer is used to unify parameter magnitudes, which accelerates convergence and prevents network overfitting, with the expression as follows

(9) $$x_{i,k}^{\left(l+1\right)*}=\gamma \frac{x_{i,k}^{l*}-\mu }{\sqrt{\sigma^{2}+\varepsilon }}+\beta$$

(10) $$\mu =\frac{1}{m}\sum_{i=1}^{m}x_{i,k}^{l*}$$

(11) $$\sigma^{2}=\frac{1}{m}\sum_{i=1}^{m}(x_{i,k}^{l*}-\mu {)}^{2}$$

where *m* represents the number of samples computed at each iteration, which is 5 in this paper; *µ* is the sample mean; *σ*^2^ is the sample variance; $x_{i,k}^{l*}$ represents the *i*-th data point of the *k*-th channel in the *l*-th layer of the model, *k*∈{1, 2, …, *N*}. The output data size of this layer remains unchanged. *γ* and *β* are, respectively, the scale and shift parameters, which can be learned through the network; *ε* is to prevent the denominator in (9) from being zero.

The rectified linear unit (ReLU) activation function is used for non-linearity processing [25], which helps alleviate the problem of gradient vanishing and is relatively simple to implement. ReLU can be expressed as follows

(12) $$x_{i,k}^{\left(l+1\right)*}=\max(x_{i,k}^{l*},0)$$

The output data size of this layer is 24 × 125 × 1. Finally, the features are dimensionally reduced through a maximum pooling (MaxPool) layer [26] with a stride of 2, with the expression as follows

(13) $$x_{i,k}^{\left(l+1\right)*}=\max(x_{i,k}^{l*},x_{i+1,k}^{l*},\ldots ,x_{i+L_{\mathrm{MaxPool}}-1,k}^{l*})$$

where *L*_MaxPool_ represents the window length of the MaxPool, which is 3 in this paper. The output size of this layer is 24 × 63 × 1. The above steps complete the preliminary extraction of current feature information.

**Figure 7:**
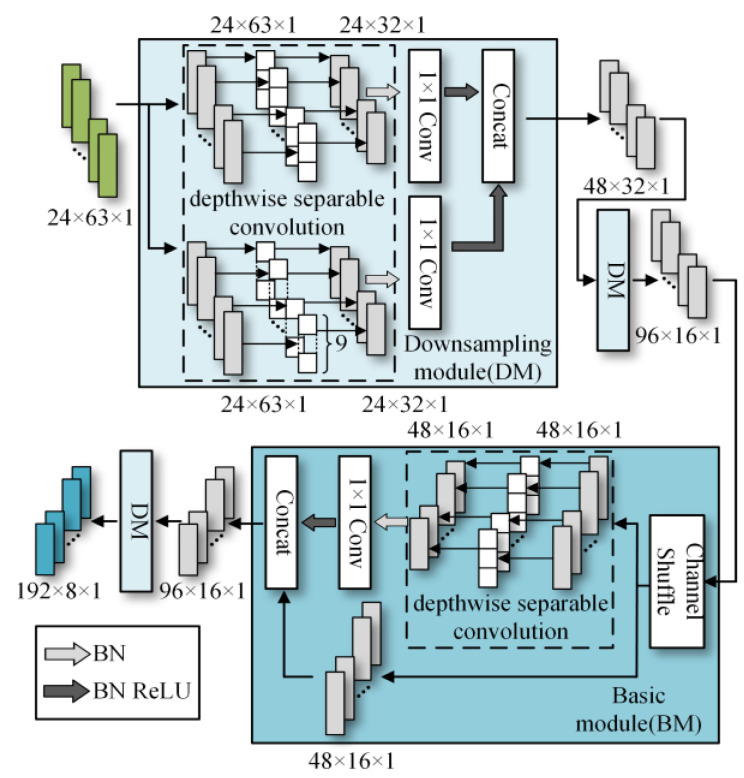
Feature depth extraction structure.

To better distinguish open-circuit faults caused by different switching device damages, it is necessary to extract higher-dimensional current fault feature information. CNN models like ResNet and DenseNet exhibit excellent performance in fields such as image recognition and object detection, but these models often have high complexity. To achieve a lightweight network model while maintaining high accuracy, rapid diagnosis and strong noise resistance in the open-circuit fault diagnosis of three-level NPC inverters, this paper designs the MSSCNN basic module and downsampling module. These modules mainly include 1 × 1 convolution layers, 3 × 1 and 9 × 1 depthwise separable convolution layers, BN layer, ReLU activation function and channel shuffle. The overall structure is shown in Figure 7.

Table 5 presents a comparison of the MSSCNN basic module and downsampling module designed in this paper with four other common CNN models. In traditional CNN architectures, each layer extracts input feature information through ordinary convolutions, but the learning capacity of features is insufficient. In ResNet, low-level feature information is directly mapped to high-level networks through short connections, which greatly improves the convergence speed and accuracy of the network. However, the large number of addition operations in the network leads to high computational complexity. ShuffleNet V2 is a lightweight network that replaces addition operations in ResNet with concat operations, thereby reducing the model’s computational load. MobileNet V3 replaces ordinary convolutions with depthwise separable convolution [27] to reduce the computational load while maintaining good classification performance. The basic modules and downsampling modules designed in this paper use depthwise separable convolutions instead of standard convolutions in traditional CNNs. Compared to ShuffleNet V2 and MobileNet V3, to reduce computation, 1 × 1 convolutions are omitted before applying depthwise separable convolutions. Moreover, concat operations replace addition operations in ResNet and MobileNet V3 to further decrease the computational complexity. In the downsampling module, a combination of 3 × 1 convolutions and 9 × 1 large kernel convolutions effectively extracts current fault features at various scales, enhancing the model’s robust fault feature extraction capabilities.

The depthwise separable convolution adopted by the proposed model in this paper is divided into two steps: depthwise convolution and pointwise convolution. As shown in Figure 7, each input channel is convolved by only one convolution kernel, so the number of output channels is exactly equal to the number of channels in the previous layer. Since each channel is convolved separately, the features in the channel direction are independent. Therefore, the second step of depthwise separable convolution is to use pointwise convolution, specifically 1 × 1 convolution, to fuse cross-channel information. The computational multiplication of standard convolution can be expressed as follows

(14) $$c_{\mathrm{standard}}=K\cdot K\cdot M\cdot F\cdot F\cdot N$$

where *K* and *F* are the sizes of the convolution kernel and the output feature map, respectively; *M* and *N* are the number of input channels and output channels, respectively.

The computational multiplication of depthwise separable convolution can be expressed as follows

(15) $$c_{\mathrm{depth}}=K\cdot K\cdot M\cdot F\cdot F+F\cdot F\cdot M\cdot N$$

The computational complexity of standard convolution and depthwise separable convolution can be compared as follows

(16) $$\frac{c_{\mathrm{depth}}}{c_{\mathrm{standard}}}=\frac{K\cdot K\cdot M\cdot F\cdot F+F\cdot F\cdot M\cdot N}{K\cdot K\cdot M\cdot F\cdot F\cdot N}=\frac{1}{N}+\frac{1}{K^{2}}$$

According to (16), it is evident that the computational complexity of the depthwise separable convolution used in this paper is significantly reduced compared to standard convolution.

As shown in Figure 7, the first step in feature depth extraction is to use a downsampling module to reduce dimensionality and extract information. The current feature information with an input size of 24 × 63 × 1 is convolved by 3 × 1 depthwise separable convolution and 9 × 1 depthwise separable convolution, respectively. This enables the network to capture current information features at different scales. During the pointwise convolution process, the input and output channels remain the same because having similar input and output channel counts minimizes memory usage and speeds up computation. After the convolutions, the two branches are concatenated, doubling the output channels and significantly enhancing the feature learning capability of the network. Another downsampling module is then used to extract feature information with an output size of 96 × 16 × 1.

In the basic module, the feature information is first subjected to channel shuffle. Channel shuffle [22] involves randomly dividing the feature channels into two groups, with each group having half the number of input feature channels. The feature channels in one branch go directly to the next layer without any operation, thereby establishing connection relationships between different layers, allowing each layer to reuse half of the features from the previous layer. This characteristic, similar to DenseNet, contributes to the model’s high accuracy. The other branch uses 3 × 1 depthwise separable convolution. The outputs of these two branches are concatenated while maintaining the channel count at 96. The final high-dimensional information feature extraction is accomplished using the downsampling module again.

**Figure 8:**
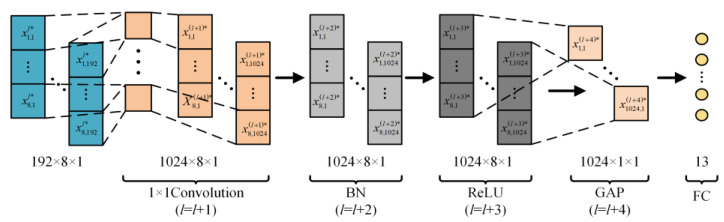
Feature summary and output.

The final step in feature depth extraction involves a simple concatenation of the two branches. Therefore, in the feature aggregation and output part, 1 × 1 convolution is first used to enhance information exchange between the two branches, as shown in Figure 8. To better distinguish the total of 13 conditions, including various types of single-switch open-circuit faults and normal operation in the three-level NPC inverter, a global average pooling (GAP) layer [26] is utilized to integrate the global information of the features, with the expression as follows

(17) $$x_{i,k}^{\left(l+1\right)*}=\frac{1}{n_{k}}\sum_{i=1}^{n_{k}}x_{i,k}^{l*}$$

where *n_k_* represents the total amount of data in the *k*-th channel. Finally, the 13 operating states are output through a fully connected (FC) layer.

## 4. Experimental Verification

### 4.1. Experimental Platform

**Figure 9:**
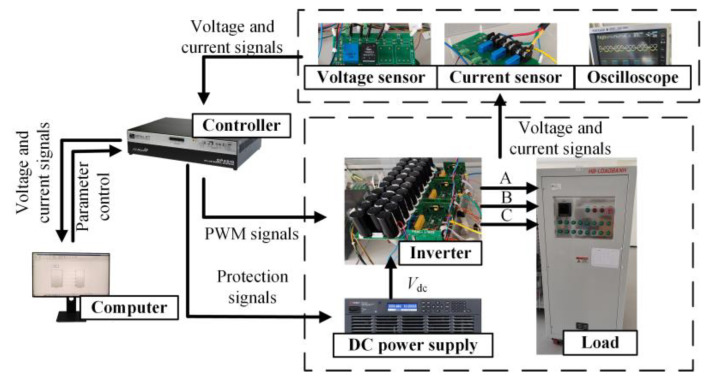
Experimental platform.

The development and training of the fault diagnosis model and the experimental platform for data detection and collection are shown in Figure 9. An OP4510 real-time simulator from OPAL-RT Technologies is used as the controller. The inverter employed in this study is FSD Company’s FPS015TI072LA001. The DC power supply, RP7972A, supplies a 150 V DC side voltage. The load comprises a resistive-inductive configuration with 22 Ω resistance and 4 mH inductance. The switching frequency is 2 kHz.

### 4.2. Data Acquisition

Nine different operating conditions are chosen for validating the effectiveness of the proposed algorithm. These conditions are determined by selecting modulation indices of 0.3, 0.6 and 0.9, as well as fundamental frequencies of 20 Hz, 30 Hz and 50 Hz. There are 13 kinds of single-switch open-circuit faults and normal operation experiments under each working condition. In these experiments, open-circuit fault situations are simulated by deactivating the driving signals to the power switching devices. The three-phase current output of the three-level NPC inverter is sampled at a frequency of 2.5 GHz using a YOKOGAWA oscilloscope and a YOKOGAWA 701932 current probe manufactured by the Yokogawa Electric Corporation in Tokyo, Japan, with a bandwidth of 100 MHz and a maximum input of 30 Arms. The collected current data are compiled into a dataset based on the content of the third section. The allocation of the training and test sets is shown in Table 6, comprising a total of 1170 test samples and 5460 training samples. The size of each sample is 3 × 250 × 1.

**Figure 10:**
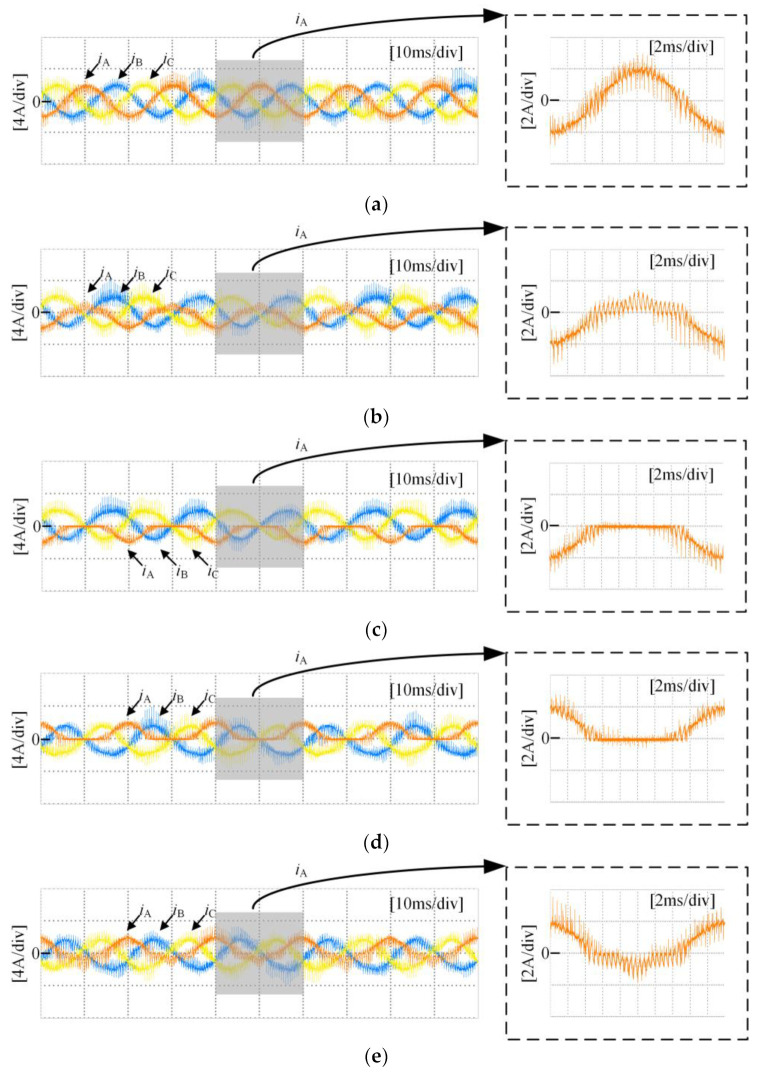
Three-phase current under open-circuit condition: (**a**) no fault condition; (**b**) S_A1_ fault; (**c**) S_A2_ fault; (**d**) S_A3_ fault; (**e**) S_A4_ fault.

Figure 10 shows the three-phase current waveforms under a no-fault condition and when each power switching device in phase A undergoes an open-circuit fault, at a 0.6 modulation index and 50 Hz fundamental frequency. As shown in Figure 10b, it can be observed that when S_A1_ experiences an open-circuit fault, the negative half-cycle current of the faulty phase is unaffected because current can flow through the anti-parallel diode on S_A1_ in the P state. However, the positive half-cycle current is distorted due to the open-circuit fault of S_A1_, as in the P state, the open device causes the forward current to only flow from D_A1_ and S_A2_ towards the load, changing the actual state from P to O state. As shown in Figure 10c, it can be seen that when S_A2_ experiences an open-circuit fault, the negative half-cycle current of the faulty phase is unaffected, as current can flow through the anti-parallel diode on S_A2_ in either the P or O state. However, the forward current is nearly zero because in the P or O state, current can only flow from the anti-parallel diodes on S_A3_ and S_A4_ towards the load, resulting in a change in the actual state to an N state. Some experimental data can be found in Table S1 of Supplementary Materials.

### 4.3. Analysis and Comparison of Diagnostic Effect

**Figure 11:**
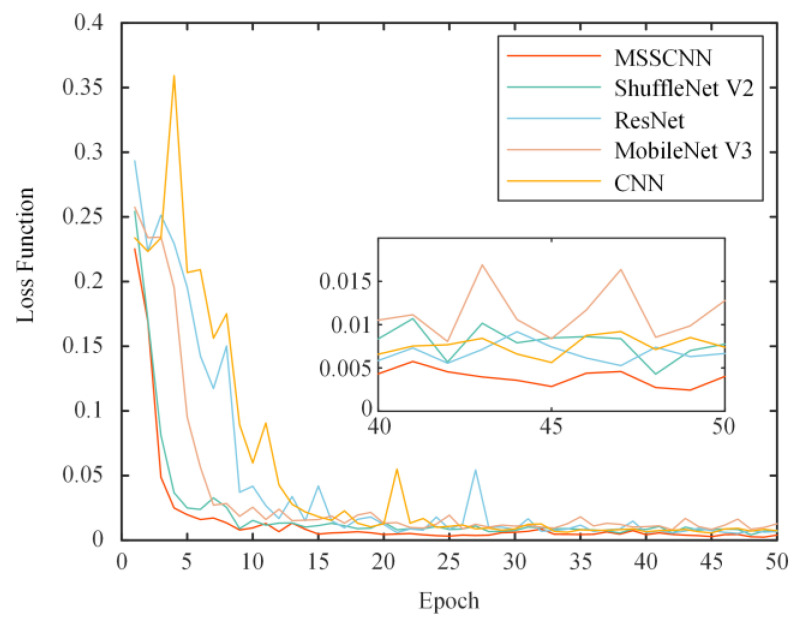
Comparison of the loss function.

To demonstrate the superior performance of the proposed algorithm, fault diagnosis models for CNN, ResNet, ShuffleNet V2 and MobileNet V3 are established based on Table 5. The training set is subsequently input into the MSSCNN, classical CNN, ResNet, ShuffleNet V2 and MobileNet V3 models for training, and the test set is used to validate the training performance of the models. The experiment is repeated 10 times using the same training and test sets, and the loss function comparison curves from one of the experiments are shown in Figure 11.

From Figure 11, it can be observed that the MSSCNN model has a faster reduction rate of loss and a lower loss value compared to the other network models, indicating that the proposed network model in this paper converges faster and has higher accuracy.

**Figure 12:**
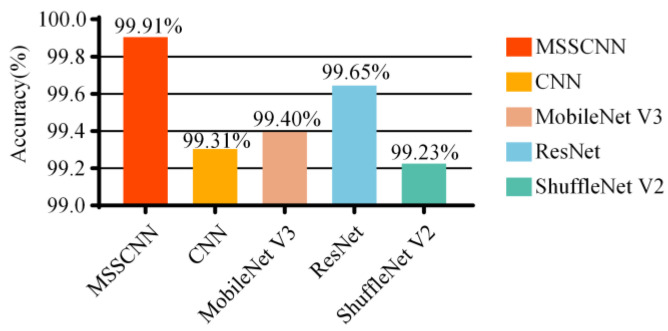
Comparison of accuracy of different models.

Figure 12 shows the average accuracy of different models based on the results of 10 experiments. The MSSCNN model achieves an accuracy of 99.91%, which is the highest among the five network models.

**Figure 13:**
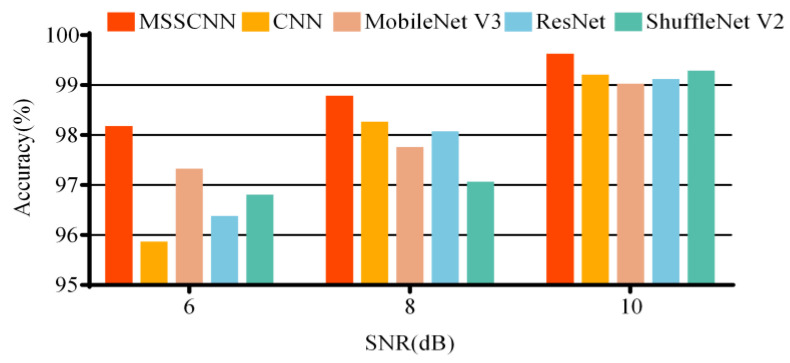
Diagnostic accuracy of each model in different noise environments.

The diagnostic effectiveness of the models in different noise environments is tested by adding noise with varying signal-to-noise ratios (SNRs) to the collected current data. The average accuracy from 10 repeated experiments is shown in Table 7 and Figure 13. It can be observed that the diagnostic accuracy of the model gradually decreases with the increase in noise intensity. The fault diagnosis network model proposed in this paper outperforms the other four models in diagnostic effectiveness under different noise environments.

**Figure 14:**
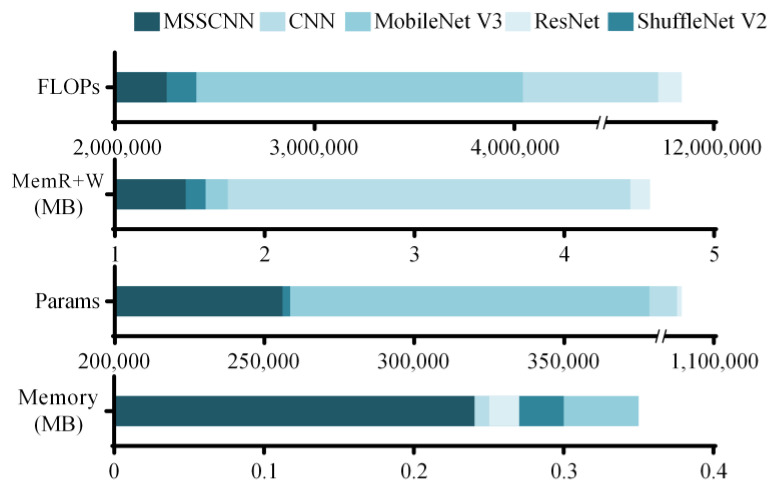
Complexity comparison of different models.

Parameters (Params), floating-point operations (FLOPs), memory required during node inference (memory) and the size of memory read and write operations (MemR + W) serve as crucial metrics for evaluating model complexity. Table 8 and Figure 14 display these metrics for various network models when applied to a 3 × 250 × 1 fault sample. The MSSCNN model demonstrates lower values across all four parameters compared to the other four network models, indicating lower complexity.

**Figure 15:**
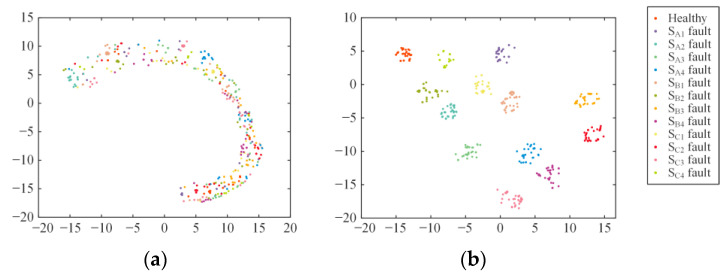
Data feature distribution visualization: (**a**) Feature distribution of input data. (**b**) Feature distribution of MSSCNN model’s output data.

To better demonstrate the classification effectiveness of the fault diagnosis model proposed in this paper, the t-SNE method is utilized for dimensionality reduction and visualization of the data in the test samples [28], as shown in Figure 15. It can be observed that the input data features shown in Figure 15a are chaotic in distribution. After being processed by the MSSCNN model, the 13 types of open-circuit faults in the three-level NPC inverter in Figure 15b are separated, with virtually no overlap of different category samples. This indicates that the model possesses excellent feature extraction capability.

## 5. Conclusions

This study proposes a fault diagnosis method for three-level NPC inverters based on the MSSCNN model and validates its effectiveness on an experimental platform. Compared to prior works like references [12,16], this method can identify power switching device open-circuit faults without the need for complex preprocessing such as wavelet packet transforms or Clark transforms on the original three-phase current data. The basic modules and downsampling modules in the MSSCNN model utilize depthwise separable convolution and channel shuffle techniques, ensuring both efficient feature extraction and a lightweight model structure. The combination of convolution kernels of different sizes enhances the model’s noise resistance. The fault diagnosis method based on the MSSCNN model proposed in this paper attained an accuracy rate of 99.91% in detecting open-circuit faults in power switching devices. Furthermore, it has higher accuracy than the comparative models built on CNN, ResNet, ShuffleNet V2 and Mobilenet V3 in three noise environments of 6 dB, 8 dB and 10 dB. Meanwhile, it has lower model complexity and surpasses the four comparative models in terms of Params, FLOPs, memory and MemR + W. Through processes such as feature enhancement and effective extraction, the proposed model can effectively improve fault determination and localization capabilities by clustering the originally mixed data feature distributions.

## Figures and Tables

**Table 1:** Output voltage and switching states of three-level NPC inverter.

Switching State	S*_x_*_1_	S*_x_*_2_	S*_x_*_3_	S*_x_*_4_	Output Voltage *v_x_*_o_
P	1	1	0	0	*V*_dc_/2
O	0	1	1	0	0
N	0	0	1	1	−*V*_dc_/2

**Table 2:** Output voltage variation before and after open-circuit fault.

*v_x_*_o_ Variation		P		O		N	
		*i_x_* ≥ 0	*i_x_* < 0	*i_x_* ≥ 0	*i_x_* < 0	*i_x_* ≥ 0	*i_x_* < 0
Fault IGBT (S)	S*_x_*_1_	*V*_dc_/2→0	--	--	--	--	--
	S*_x_*_2_	*V*_dc_/2→−*V*_dc_/2	--	0→−*V*_dc_/2	--	--	--
	S*_x_*_3_	--	--	--	0→*V*_dc_/2	--	−*V*_dc_/2→*V*_dc_/2
	S*_x_*_4_	--	--	--	--	--	−*V*_dc_/2→0

**Table 3:** Three-phase current waveforms of each power switching device in phase A under open-circuit fault.

Fault IGBT (S)	Three-Phase Current Waveforms
S_A1_	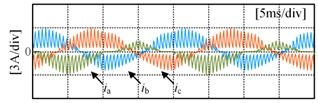
S_A2_	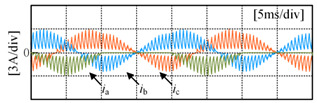
S_A3_	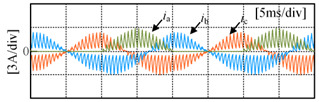
S_A4_	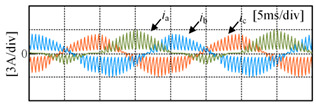

**Table 4:** Fault classification.

Fault IGBT (S)	Label	Fault IGBT (S)	Label	Fault IGBT (S)	Label
None	0	S_B1_	5	S_C1_	9
S_A1_	1	S_B2_	6	S_C2_	10
S_A2_	2	S_B3_	7	S_C3_	11
S_A3_	3	S_B4_	8	S_C4_	12
S_A4_	4				

**Table 5:** Comparison of structure between the basic module and downsampling module.

Model	Basic Modules and Downsampling Modules
MSSCNN (Model of this paper)	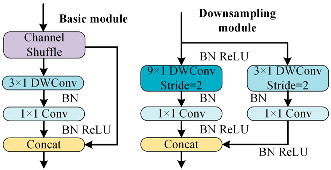
CNN [20]	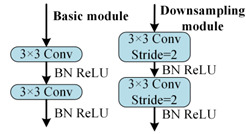
ResNet [21]	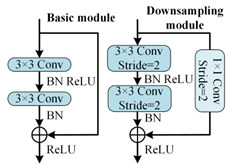
ShuffleNet V2 [22]	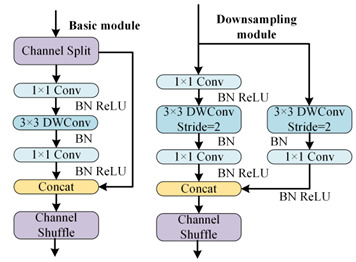
Mobilenet V3 [23]	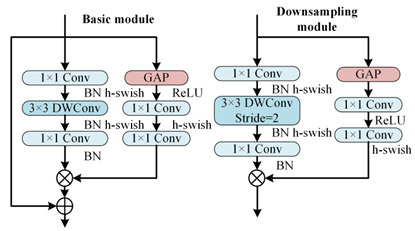

**Table 6:** Division of training set and test set.

Data Set	Modulation Index	Fundamental Frequency (Hz)	Sample Number
Test set	0.9	50	390
	0.6	30	390
	0.3	20	390
Training set	0.9	30	910
	0.9	20	910
	0.6	50	910
	0.6	20	910
	0.3	50	910
	0.3	30	910

**Table 7:** Diagnostic accuracy of each model in different noise environments.

SNR (dB)	Accuracy (%)				
	MSSCNN	CNN	ResNet	MobileNet V3	ShuffleNet V2
6	98.20	95.89	87.26	97.35	96.83
8	98.81	98.29	98.80	97.78	97.09
10	99.65	99.23	99.14	99.05	99.31

**Table 8:** Complexity comparison of different models.

Model	Params	FLOPs	Memory	MemR + W
MSSCNN	256,248	2,264,136	0.24 MB	1.44 MB
CNN	1,034,328	11,382,600	0.25 MB	4.38 MB
ResNet	1,058,856	11,640,648	0.27 MB	4.71 MB
MobileNet V3	378,864	4,049,256	0.35 MB	1.76 MB
ShuffleNet V2	258,984	2,413,992	0.30 MB	1.58 MB

## Data Availability

Data are contained within the article.

## Supplementary Materials

The following supporting information can be downloaded at: https://www.mdpi.com/article/10.3390/s24061745/s1, Table S1: Three-phase current data.
