# Supplementary material for: An Open-Circuit Fault Diagnosis Method for Three-Level Neutral Point Clamped Inverters Based on Multi-Scale Shuffled Convolutional Neural Network
Source: Sensors (Basel). 2024 Mar 7;24(6):1745. doi: 10.3390/s24061745 (PMC10975164; doi:10.3390/s24061745)
Supplement: Supplementary file 1 [file sensors-24-01745-s001.zip › sensors-2865392-supplementary.pdf]

sensors

MDPI

Supplementary Materials for

An Open-Circuit Fault Diagnosis Method for Three-Level Neutral Point Clamped Inverters Based on Multi-Scale Shuffled Convolutional Neural Network

The three-phase current data for the NPC inverter is provided. These data are obtained when a single-device open circuit fault of the A-phase arm occurs. Experimental conditions: Modulation index of 0.6, Output frequency of 50 Hz, Resistive-inductive load of 22 Ω/4 mH, Switching frequency of 2 kHz.Due to space constraints, the data consists of one fundamental cycle, with a total of 250 data points.

Table S1. Three-phase current data.

Fault IGBT (S)	Current Data
SA1	Phase A: −0.7468003, −0.9374062, −0.4218328, −0.4218328, 0.149985, −0.5395294, −0.8311669, −0.3957937, 0.1093641, 0.4426641, −0.3739209, −0.4770356, −0.1364447, 0.2874713, 0.5853581, 0.01562344, −0.1635253, −0.06249375, 0.3864197, 0.63327, −0.00416625, −0.02812219, 0.13332, 0.4051678, 0.6030647, −0.06561844, −0.02083125, 0.2645569, 0.549945, 0.6957638, −0.005207812, −0.03853781, 0.2239359, 0.5874413, 0.6718078, 0.13332, 0.06249375, 0.2041463, 0.5791088, 0.7655484, 0.3416325, 0.183315, 0.3208013, 0.7957538, 0.8061694, 0.4239159, 0.2343516, 0.3468403, 0.9988585, 0.9655284, 0.5009916, 0.2343516, 0.3968353, 1.084267, 1.125929, 0.592649, 0.2968453, 0.5551528, 1.098848, 1.19988, 0.6478519, 0.2885128, 0.5134903, 1.072809, 1.156134, 0.46662, 0.2905959, 0.43329, 1.005108, 1.03323, 0.5197397, 0.2426841, 0.4614122, 0.7707562, 0.8061694, 0.3707963, 0.1374862, 0.3770456, 0.5947322, 0.7655484, 0.3197597, −0.03645469, 0.2426841, 0.5343215, 0.7311769, 0.006249375, −0.05311969, 0.2343516, 0.5655684, 0.6207712, −0.007290937, −0.016665, 0.1781072, 0.5707762, 0.6541013, −0.06666, −0.05624437, 0.08228344, 0.4187081, 0.59994, −0.04270406, −0.2114372, −0.09686531, 0.3687131, 0.6613922, −0.2208112, −0.4572459, −0.1770656, 0.3030947, 0.5374463, −0.3010116, −0.6957638, −0.2687231, −0.39996, 0.1947722, −0.4687031, −0.8572059, −0.3853781, −0.6134803, −0.2270606, −0.7457588, −0.9894844, −0.7072209, −0.8342916, −0.6790987, −0.9894844, −1.011357, −1.040521, −1.048853, −1.234252, −1.26654, −1.200922, −1.426941, −1.200922, −1.507141, −1.335283, −1.330075, −1.511307, −1.4134, −1.710246, −1.426941, −1.367572, −1.468603, −1.708163, −1.842524, −1.529014, −1.698788, −1.697747, −1.830025, −1.809194, −1.857106, −1.967512, −1.843566, −1.865438, −1.781072, −1.89981, −2.145619, −1.871688, −1.888353, −1.798778, −1.953971, −2.250817, −1.815443, −1.931057, −1.911267, −1.916475, −2.084167, −1.842524, −1.885228, −1.868563, −1.683165, −1.700872, −1.706079, −1.792529, −1.811277, −1.557136, −1.45298, −1.481102, −1.43319, −1.63317, −1.502975, −1.343616, −1.494642, −1.270706, −1.450897, −1.382153, −1.293621, −1.41965, −1.197797, −1.122804, −1.162384, −1.234252, −0.9144919, −0.9103256, −0.6905559, −0.8842866, −1.03323, −0.4947422, −0.7520081, −0.2291438, −0.7311769, −0.9478219, −0.4291238, −0.4239159, 0.1864397, −0.5978569, −0.8072109, −0.3520481, 0.1041562, 0.4020431, −0.3937106, −0.5145319, −0.1583175, 0.3260091, 0.5509866, −0.05103656, −0.1593591, −0.02708063, 0.349965, 0.607231, −0.01458187, −0.02395594, 0.1343616, 0.4114172, 0.542654, −0.04895344, 0.0083325, 0.2645569, 0.5009916, 0.7249275, −0.007290937, −0.01874813, 0.2905959, 0.5957738, 0.6863897, 0.1354031, 0.02187281, 0.2687231, 0.5384878, 0.6947222, 0.3791288, 0.1645669, 0.3010116, 0.7645069, 0.8197097, 0.3926691, 0.2426841, 0.3353831, 0.9551128<br>Phase B: −1.312369, −1.226961, −1.441522, −1.347782, −1.551928, −1.410276, −1.388403, −1.522764, −1.588383, −1.774822, −1.467562, −1.48006, −1.567552, −1.761282, −1.78628, −1.626921, −1.65296, −1.654001, −1.798778, −1.816485, −1.629004, −1.798778, −1.588383, −1.792529, −1.669625, −1.58005, −1.655043, −1.500892, −1.728994, −1.771698, −1.29987, −1.543596, −1.382153, −1.640461, −1.682123, −1.130095, −0.9676116, −1.056144, −1.438398, −1.607131, −0.8384578, −0.5176566, −0.8530397, −1.0801, −1.477977, −0.7801303, −0.4270406, −0.7384678, −0.8936606, −1.276956, −0.6874313, −0.3947522, −0.6686831, −0.7811719, −0.9894844, −0.5176566, −0.3145519,

	−0.4270406, −0.6582675, −0.5988984, −0.3968353, −0.1989384, −0.01145719, −0.4593291, −0.083325, −0.09061594, −0.005207812, 0.2010216, −0.1531097, 0.2031047, 0.08228344, 0.0083325, 0.2239359, 0.2458088, 0.5895244, 0.2103956, 0.2458088, 0.3843366, 0.6103556, 0.6811818, 0.4853681, 0.823876, 0.7218028, 0.8342916, 0.8249175, 1.041563, 1.389444, 0.9696947, 0.9592791, 0.9217828, 1.361322, 1.572759, 1.322784, 1.121763, 0.9644869, 1.435273, 1.640461, 1.558177, 1.330075, 1.105098, 1.606089, 1.820651, 1.640461, 1.292579, 1.163425, 1.796695, 1.907101, 1.707121, 1.337366, 1.157176, 1.791487, 2.048753, 1.841483, 1.669625, 1.349865, 1.849815, 2.106039, 1.840441, 1.820651, 1.593591, 1.95293, 2.15291, 1.844607, 1.853981, 1.630045, 1.987301, 2.194572, 1.851898, 1.861272, 1.629004, 1.958138, 2.187281, 1.840441, 1.856064, 1.496725, 1.98001, 2.106039, 1.81961, 1.726911, 1.291538, 1.815443, 2.085208, 1.816485, 1.458187, 1.158218, 1.759199, 1.789404, 1.642544, 1.307161, 1.092599, 1.491518, 1.506099, 1.48631, 1.21967, 1.065518, 1.179049, 1.136345, 1.039479, 0.9780272, 0.8697047, 0.890536, 1.056144, 0.6749325, 0.7936707, 0.9217828, 0.5228644, 0.5561944, 0.3749625, 0.5676516, 0.7311769, −0.07603406, −0.1541512, −0.05416125, 0.3843366, 0.6416025, −0.23331, −0.6978469, −0.2822634, −0.3103856, 0.2978869, −0.4343316, −0.8113772, −0.3582975, −0.5384878, −0.08228344, −0.6061894, −0.9196997, −0.5988984, −0.7634653, −0.5655684, −0.8311669, −1.043646, −0.9592791, −0.9696947, −0.96657, −1.054061, −1.170716, −1.376946, −1.187381, −1.358197, −1.369655, −1.268623, −1.456104, −1.363405, −1.623796, −1.423816, −1.367572, −1.506099, −1.611297, −1.740451, −1.490476, −1.522764, −1.543596, −1.767532, −1.769615, −1.662334, −1.697747, −1.702955, −1.768573, −1.836275, −1.692539, −1.744617, −1.582133, −1.706079, −1.605048, −1.530055, −1.638378, −1.507141, −1.716495, −1.797737, −1.398818, −1.588383, −1.347782, −1.597757, −1.732118, −1.093641, −0.9999, −1.020731, −1.435273, −1.600882, −0.8780372, −0.4676616, −0.8103356, −1.190506, −1.468603, −0.7624238, −0.4145419, −0.6874313, −0.9196997, −1.281122<br>Phase C: 1.948763, 2.16645, 1.838358, 1.684207, 1.398818, 1.867522, 2.070626, 1.857106, 1.483185, 1.236335, 1.855023, 1.947722, 1.727952, 1.370696, 1.134262, 1.625879, 1.811277, 1.629004, 1.287371, 1.104056, 1.609214, 1.698788, 1.457146, 1.154051, 1.067602, 1.496725, 1.641502, 1.235293, 1.122804, 1.020731, 1.281122, 1.558177, 1.061352, 0.93324, 0.9426141, 0.8613722, 0.9082425, 0.6874313, 0.823876, 0.8915775, 0.416625, 0.3426741, 0.4603706, 0.2895544, 0.6145219, 0.2968453, 0.1656084, 0.2791387, −0.1114472, 0.3187181, 0.09894843, 0.1145719, 0.2520581, −0.3103856, −0.1437356, −0.09582375, −0.05624437, −0.1676916, −0.5395294, −0.6728494, −0.3770456, −0.1541512, −0.4937006, −0.6780572, −1.051978, −0.4728694, −0.3197597, −0.7197197, −0.9009516, −1.229044, −0.6291038, −0.3083025, −0.7530497, −1.114472, −1.459229, −0.69993, −0.46662, −0.7749225, −1.218628, −1.483185, −0.8519982, −0.8822035, −1.026981, −1.379029, −1.643586, −1.110306, −1.418608, −1.298828, −1.545679, −1.584217, −1.35299, −1.556094, −1.502975, −1.716495, −1.6665, −1.479019, −1.611297, −1.656084, −1.736285, −1.797737, −1.631087, −1.584217, −1.64671, −1.740451, −1.774822, −1.482143, −1.53318, −1.638378, −1.71337, −1.740451, −1.496725, −1.334242, −1.579009, −1.350907, −1.620671, −1.34674, −1.287371, −1.521723, −1.226961, −1.340491, −1.308203, −1.331117, −1.196755, −1.025939, −1.005108, −1.015523, −1.196755, −0.8249175, −0.8811619, −0.4259991, −0.8613722, −1.049895, −0.4614122, −0.607231, −0.1031147, −0.657226, −0.9144919, −0.4645369, −0.3905859, 0.3020531, −0.4072509, −0.8009616, −0.3749625, 0.216645, 0.4759941, −0.3333, −0.19998, 0, 0.3385078, 0.6186881, 0.2083125, 0.4020431, 0.3270506, 0.5468203, 0.6353531, 0.6915975, 0.86658, 0.7811719, 0.8301253, 0.7353431, 1.009274, 1.088433, 1.039479, 0.9551128, 0.9519881, 1.3332, 1.509224, 1.422774, 1.225919, 1.075934, 1.689414, 1.789404, 1.624838, 1.329034, 1.137386, 1.791487, 2.028964, 1.76649, 1.660251, 1.279039, 1.853981, 2.090416, 1.73316, 1.798778, 1.479019, 1.942514, 2.174783, 1.875854, 1.889394, 1.598798, 1.962304, 2.153951, 1.847732, 1.857106, 1.65296, 1.989384, 2.143536, 1.824818, 1.831067, 1.520681, 2.041462, 2.135203, 1.839399, 1.790446, 1.379029, 1.931057, 2.15291, 1.845649, 1.443606, 1.257166, 1.858148, 1.962304, 1.728994, 1.322784, 1.135303, 1.63317, 1.845649, 1.637336, 1.269665, 1.124887, 1.64671, 1.687331, 1.401943, 1.195714, 1.001983, 1.524848, 1.611297, 1.232168, 1.105098, 1.011357, 1.371738, 1.560261, 1.016565, 1.025939, 0.9894844, 0.8384578, 0.8780372, 0.6926391, 0.8217928, 0.8561644, 0.416625, 0.283305, 0.4270406, 0.3780872, 0.6541013, 0.3228844, 0.149985, 0.2916375, −0.1124887, 0.2676816
SA2	Phase A: −0.016665, 0.01249875, −0.0083325, 0.04582875, 0.01354031, −0.03437156, −0.02291437, −0.06666, −0.06561844, −0.04062094, 0.01458187, 0.007290937, −0.01145719, −0.03228844, −0.01770656, −0.04895344, −0.01145719, −0.06249375, −0.03853781, −0.0249975, −0.01354031, −0.04687031, 0.02395594, 0.01249875, −0.05207812, −0.01145719, −0.03333, −0.05624437, 0.006249375, −0.05207812, −0.04270406, −0.04582875, −0.06353531, 0.0583275, 0.0583275, −0.06666, −0.04687031, −0.02083125, 0.06145219, −0.016665, −0.03853781, −0.04374563, −0.016665, −0.016665, −0.02812219, −0.03124687, −0.04062094, −0.05728594, −0.06457687, −0.01562344, −0.06041063, −0.02708063, −0.06249375, −0.1843566, −0.06457687, −0.05936906, −0.3707963, −0.05520281, −0.2989284, −0.03333, −0.01041562, −0.4259991, −0.4353731, −0.5041162, −0.016665, −0.03957938,

−0.4447472, −0.7686731, −0.6884728, −0.149985, −0.016665, −0.4541213, −0.773881, −0.7218028, −0.5041162, −0.6541013, −0.5968153, −0.8290837, −0.8342916, −0.7468003, −0.9186581, −0.8301253, −1.018648, −0.9821934, −1.006149, −1.010316, −1.210296, −1.148843, −1.274873, −1.285288, −1.463395, −1.395694, −1.297787, −1.367572, −1.54672, −1.673791, −1.585258, −1.379029, −1.451938, −1.708163, −1.844607, −1.63317, −1.801903, −1.564427, −1.810236, −1.963345, −1.725869, −1.977927, −1.739409, −1.924808, −1.958138, −1.736285, −2.003966, −1.76649, −1.903976, −1.990426, −1.856064, −2.087291, −1.737326, −1.921683, −1.991467, −1.763365, −2.021673, −1.826901, −1.91335, −1.958138, −1.741493, −1.851898, −1.540471, −1.815443, −1.91335, −1.668583, −1.411317, −1.383195, −1.64671, −1.687331, −1.645669, −1.355073, −1.385278, −1.420691, −1.356114, −1.462354, −1.324867, −1.31341, −1.235293, −1.263415, −1.175924, −1.148843, −0.9519881, −0.9769856, −0.975944, −0.8426241, −0.9894844, −0.9217828, −0.7322184, −0.5822334, −0.5739009, −0.8655384, −0.7770056, −0.2624738, −0.01249875, −0.3989184, −0.7895043, −0.7936707, −0.007290937, 0.01978969, −0.3728794, −0.4437056, −0.5509866, −0.06041063, −0.01562344, −0.4520381, −0.03957938, −0.3312169, −0.0583275, −0.001041563, −0.1458188, −0.016665, −0.08644969, −0.01978969, 0.05416125, −0.02291437, 0.003124688, −0.02187281, −0.02291437, −0.01458187, −0.03020531, −0.01145719, 0.0249975, −0.05311969, −0.03020531, −0.05207812, −0.02395594, −0.03020531, −0.02603906, −0.02395594, −0.01562344, −0.06561844, −0.02603906, −0.00416625, −0.04374563, −0.02603906, −0.005207812, −0.03645469, −0.03645469, −0.06666, −0.05936906, −0.03020531, −0.06041063, −0.0416625, −0.0249975, −0.03645469, 0.03541312, −0.06145219, 0.0416625, −0.04374563, −0.06561844, −0.03124687, −0.01978969, −0.04270406, −0.06145219, −0.03437156, −0.01041562, −0.03124687, −0.02708063, −0.0416625, −0.007290937, −0.03020531, −0.02291437, −0.05103656, −0.02083125, −0.03541312, −0.01978969, −0.01145719, −0.06561844, −0.04582875, −0.05936906, 0.06249375, −0.001041563, −0.0249975, −0.04270406, −0.007290937, −0.002083125, 0.009374063, −0.002083125, −0.01354031, −0.03541312, −0.04687031, −0.04791187, −0.01354031

Phase B: −1.016565, −0.7915875, −0.4259991, −0.3051778, −0.6363947, −0.6457688, −0.5718178, −0.2812219, −0.2989284, −0.3739209, −0.1385278, −0.2114372, −0.1385278, −0.1229044, −0.06770156, −0.03124687, 0.009374063, −0.05311969, −0.04062094, 0.1322784, 0.1718578, 0.2614322, 0.07603406, 0.07915875, 0.3333, 0.5988984, 0.4957837, 0.2187281, 0.1885228, 0.7395094, 0.93324, 0.7145119, 0.4645369, 0.2760141, 0.8342916, 1.164467, 0.7436756, 1.000942, 0.6978469, 1.009274, 1.247792, 0.9144919, 1.257166, 0.9342816, 1.247792, 1.365488, 1.304036, 1.432148, 1.338408, 1.405068, 1.461312, 1.447772, 1.568593, 1.737326, 1.581092, 1.471728, 1.785238, 1.632128, 1.85294, 1.543596, 1.591508, 1.775864, 1.814402, 2.041462, 1.584217, 1.559219, 1.772739, 1.960221, 2.016465, 1.712329, 1.559219, 1.798778, 1.938348, 2.071668, 1.809194, 1.850857, 1.853981, 2.065418, 1.89981, 1.853981, 1.915433, 1.840441, 2.053961, 1.847732, 1.865438, 1.923766, 1.900852, 2.096665, 2.117496, 1.856064, 1.772739, 1.831067, 1.98626, 1.9998, 1.579009, 1.520681, 1.618588, 1.898768, 1.970636, 1.471728, 1.327992, 1.5863, 1.471728, 1.794612, 1.408193, 1.228002, 1.559219, 1.398818, 1.554011, 1.296745, 1.14676, 1.142594, 1.123846, 1.059269, 1.039479, 0.9790688, 0.8134603, 0.9509466, 0.709304, 0.8353331, 0.9092841, 0.2645569, 0.6113972, 0.2447672, 0.5728593, 0.7436756, 0.1374862, 0.2655984, −0.2301853, 0.3280922, 0.6207712, 0.07395094, −0.5009916, −0.5489035, 0.009374063, 0.1687331, −0.1947722, −0.6416025, −0.6936806, −0.4207912, −0.4176666, −0.4312069, −0.7603406, −0.7572159, −0.7197197, −0.8061694, −0.7863797, −0.8801203, −0.8978269, −0.9561543, −1.060311, −1.041563, −1.174883, −1.137386, −1.308203, −1.273831, −1.39986, −1.293621, −1.214462, −1.531097, −1.581092, −1.516515, −1.388403, −1.277997, −1.604006, −1.558177, −1.46652, −1.514432, −1.354031, −1.607131, −1.58005, −1.44673, −1.657126, −1.417567, −1.583175, −1.573801, −1.56651, −1.642544, −1.435273, −1.487351, −1.511307, −1.458187, −1.577967, −1.34674, −1.426941, −1.497767, −1.248833, −1.445689, −1.123846, −1.394652, −1.462354, −0.990526, −1.209254, −0.69993, −1.069685, −1.269665, −0.8676215, −0.59994, −0.3905859, −0.9571959, −1.043646, −0.7113872, −0.4228744, −0.3103856, −0.6145219, −0.6197297, −0.5624437, −0.2937206, −0.2541412, −0.2697647, −0.1541512, −0.2385178, −0.09894843, −0.06770156, −0.06978469, −0.006249375, −0.06145219, −0.009374063, −0.00416625, 0.07186782, 0.1989384, 0.2031047, 0.08436656, 0.07707562, 0.3926691, 0.5718178, 0.4916175, 0.2208112, 0.1406109, 0.7967953, 0.9238659, 0.6926391, 0.4030847, 0.2343516, 0.8874112, 1.18634, 0.7530497, 1.058228, 0.6416025, 1.084267, 1.239459, 0.8197097, 1.216545, 0.9603207, 1.225919, 1.354031, 1.298828, 1.455063, 1.398818, 1.426941

Phase C: 1.000942, 0.6676416, 0.3635053, 0.2989284, 0.5707762, 0.59994, 0.5239059, 0.2603906, 0.1937306, 0.2989284, 0.1010316, 0.2270606, 0.09374063, 0.03124687, 0.05624437, −0.01562344, −0.06145219, −0.04478719, −0.01249875, −0.1551928, −0.1708162, −0.3322584, −0.1885228, −0.2447672, −0.4176666, −0.5718178, −0.5218228, −0.2416425, −0.3333, −0.8061694, −0.9780272, −0.716595, −0.4687031, −0.3426741, −0.8728294, −1.136345, −0.8551228, −1.121763, −0.7061794, −1.115513, −1.215503, −0.8697047, −1.295704, −1.030105, −1.315493, −1.373821, −1.327992, −1.481102, −1.334242, −1.514432, −1.531097, −1.526931, −1.547762, −1.547762, −1.538388,

	−1.579009, −1.461312, −1.676916, −1.538388, −1.634212, −1.569635, −1.461312, −1.491518, −1.450897, −1.650877, −1.538388, −1.451938, −1.285288, −1.348823, −1.491518, −1.636295, −1.462354, −1.281122, −1.334242, −1.394652, −1.28008, −1.356114, −1.261332, −1.075934, −1.15301, −1.073851, −1.012399, −1.092599, −0.9728194, −0.9280322, −0.96657, −0.6895144, −0.8780372, −0.79992, −0.5530697, −0.3478819, −0.4135003, −0.7436756, −0.6749325, −0.1281122, 0.09686531, −0.2614322, −0.6676416, −0.5384878, 0.1093641, 0.5155734, −0.03437156, 0.2541412, −0.1676916, 0.2822634, 0.5988984, 0.1135303, 0.5009916, 0.1489434, 0.5863997, 0.709304, 0.5603606, 0.8145019, 0.6124387, 0.8613722, 0.8842866, 0.9457387, 1.014482, 1.064477, 1.062394, 1.060311, 1.426941, 1.256124, 1.46652, 1.234252, 1.178007, 1.500892, 1.593591, 1.745659, 1.43319, 1.210296, 1.520681, 1.856064, 1.918558, 1.589424, 1.431107, 1.65296, 1.905018, 1.953971, 1.709204, 1.735243, 1.832108, 1.985218, 2.057086, 1.927932, 1.981052, 1.75295, 1.9998, 1.820651, 1.926891, 1.953971, 1.818568, 2.005008, 1.974802, 1.910226, 1.740451, 1.864397, 2.001883, 1.95293, 1.698788, 1.48006, 1.803986, 1.995634, 1.967512, 1.535263, 1.576926, 1.791487, 1.845649, 1.88627, 1.594632, 1.569635, 1.767532, 1.621713, 1.678999, 1.5863, 1.590466, 1.618588, 1.639419, 1.476936, 1.448813, 1.471728, 1.449855, 1.549845, 1.276956, 1.410276, 1.454021, 1.18009, 1.410276, 1.023856, 1.254041, 1.387361, 0.9717778, 1.148843, 0.6593091, 1.072809, 1.287371, 0.8540813, 0.549945, 0.2697647, 0.890536, 1.041563, 0.7801303, 0.3520481, 0.2156034, 0.53328, 0.6447272, 0.4853681, 0.283305, 0.1739409, 0.2853881, 0.1656084, 0.1614422, 0.1156134, 0.05416125, 0.06249375, −0.02083125, −0.04270406, −0.049995, −0.05311969, −0.183315, −0.1822734, −0.3291337, −0.1676916, −0.1958137, −0.4478719, −0.5770257, −0.5176566, −0.3228844, −0.2124788, −0.8020031, −0.9946922, −0.7988784, −0.4905759, −0.3374662, −0.9311569, −1.19988, −0.8020031, −1.069685, −0.7113872, −1.072809, −1.292579, −0.9196997, −1.272789, −1.024897, −1.289454, −1.405068, −1.267582, −1.48631, −1.356114, −1.462354
SA3	Phase A: 1.726911, 1.86648, 1.698788, 1.345699, 1.254041, 1.61338, 1.574842, 1.507141, 1.316535, 1.173841, 1.397777, 1.249875, 1.350907, 1.214462, 1.016565, 1.108222, 1.074893, 0.96657, 0.9926091, 0.9217828, 0.8519982, 0.9040763, 0.6259791, 0.7801303, 0.8811619, 0.2676816, 0.2635153, 0.2843466, 0.642644, 0.7468003, −0.03437156, −0.001041563, 0.2281022, 0.6218128, 0.7780472, −0.06666, −0.05728594, 0.2385178, −0.04582875, 0.4082925, −0.08644969, −0.03541312, 0.2770556, −0.08853281, 0.2041463, −0.02812219, −0.02395594, −0.04791187, −0.0749925, 0.02187281, −0.003124688, −0.03957938, −0.06457687, −0.001041563, −0.01458187, −0.02291437, −0.06249375, −0.01978969, −0.05624437, −0.02812219, −0.03437156, −0.01249875, −0.1135303, −0.01145719, −0.0583275, −0.03749625, −0.06666, −0.1145719, −0.06145219, −0.02603906, −0.04687031, −0.03645469, −0.04687031, −0.02187281, −0.03020531, −0.08228344, −0.03645469, −0.0416625, −0.03124687, −0.002083125, −0.06457687, −0.03020531, −0.02812219, −0.005207812, −0.01145719, −0.01562344, −0.02916375, −0.02812219, −0.03645469, −0.02916375, −0.06561844, −0.05624437, −0.0249975, −0.03645469, −0.0249975, −0.01145719, −0.03645469, −0.04478719, −0.05728594, −0.04478719, −0.06561844, −0.03020531, −0.005207812, −0.01249875, −0.03853781, −0.03749625, −0.06666, −0.02812219, −0.05936906, −0.04478719, −0.03124687, −0.05416125, −0.002083125, −0.05520281, −0.02812219, −0.03020531, −0.05416125, −0.0583275, −0.001041563, 0.03020531, −0.01041562, −0.0416625, 0.3291337, −0.08853281, 0.3176766, −0.01562344, −0.02708063, 0.2270606, −0.03645469, 0.4010016, −0.03020531, −0.01041562, 0.3030947, 0.5041162, 0.7967953, −0.01562344, −0.003124688, 0.2249775, 0.5905659, 0.7905459, 0.1989384, 0.2864297, 0.3197597, 0.6291038, 0.7947122, 0.53328, 0.8082525, 0.6103556, 0.7707562, 0.7488834, 0.8936606, 0.9613622, 0.9613622, 0.9603207, 0.9488634, 1.187381, 1.13322, 1.302995, 1.18634, 1.078017, 1.475894, 1.577967, 1.521723, 1.244667, 1.192589, 1.696705, 1.835233, 1.610256, 1.55297, 1.301953, 1.696705, 1.994592, 1.693581, 1.747742, 1.436315, 1.823776, 1.945639, 1.702955, 1.900852, 1.597757, 1.856064, 2.01334, 1.734202, 1.906059, 1.790446, 1.921683, 2.037296, 1.602965, 1.803986, 1.620671, 1.91335, 2.039379, 1.836275, 1.809194, 1.523806, 1.83315, 2.006049, 1.7467, 1.53318, 1.374863, 1.734202, 1.888353, 1.6665, 1.371738, 1.229044, 1.554011, 1.542554, 1.532138, 1.304036, 1.142594, 1.372779, 1.24675, 1.364447, 1.206129, 0.9769856, 1.06656, 1.091558, 0.9988585, 0.9634453, 0.8874112, 0.8572059, 0.9644869, 0.6374363, 0.8072109, 0.9186581, 0.3562144, 0.216645, 0.3312169, 0.542654, 0.7686731, −0.0749925, −0.0583275, 0.2208112, 0.5801503, 0.773881, −0.01770656, −0.1239459, 0.2864297, −0.05416125, 0.4655784, −0.09478219, −0.08957437, 0.3020531, −0.02291437, 0.2353931, −0.04791187, −0.04791187, −0.03437156, −0.005207812, −0.04270406<br>Phase B: −0.3280922, −0.542654, −0.2187281, 0.3416325, 0.6645169, −0.08020031, 0.1239459, 0.1531097, 0.5197397, 0.7405509, 0.3437156, 0.6905559, 0.4478719, 0.6666, 0.7395094, 0.7915875, 0.9228244, 0.9301153, 0.9655284, 0.9634453, 1.007191, 1.044687, 1.228002, 1.167592, 1.112389, 1.412359, 1.420691, 1.409234, 1.238418, 1.145719, 1.589424, 1.606089, 1.490476, 1.274873, 1.164467, 1.631087, 1.612339, 1.525889, 1.55297, 1.305078,

	1.550887, 1.636295, 1.463395, 1.561302, 1.432148, 1.601923, 1.664417, 1.408193, 1.500892, 1.398818, 1.423816, 1.589424, 1.241542, 1.393611, 1.079059, 1.364447, 1.489434, 1.005108, 1.239459, 0.8363747, 1.205088, 1.463395, 1.057186, 1.051978, 0.4812019, 1.01969, 1.274873, 0.9874012, 0.4968253, 0.2228944, 0.8780372, 0.8322085, 0.6666, 0.3957937, 0.2208112, 0.4843266, 0.3655884, 0.4697447, 0.2156034, 0.1208213, 0.1572759, 0.02812219, 0.02395594, −0.01354031, −0.04478719, −0.05728594, −0.04062094, −0.1447772, −0.1249875, −0.05728594, −0.3780872, −0.3624637, −0.5228644, −0.2510166, −0.1728994, −0.8290837, −0.8572059, −0.6978469, −0.4020431, −0.2301853, −0.9092841, −1.265498, −0.9624038, −0.9738609, −0.5103656, −1.095724, −1.343616, −1.06656, −1.195714, −0.8113772, −1.317577, −1.436315, −1.190506, −1.38007, −1.175924, −1.382153, −1.576926, −1.421733, −1.475894, −1.515473, −1.514432, −1.548803, −1.767532, −1.535263, −1.738368, −1.6665, −1.620671, −1.735243, −1.611297, −1.83315, −1.601923, −1.629004, −1.734202, −1.89981, −1.9998, −1.607131, −1.700872, −1.859189, −1.937306, −2.049795, −1.753991, −1.850857, −1.783155, −1.93314, −2.047712, −1.944597, −2.084167, −1.839399, −1.984177, −1.88627, −1.947722, −2.115413, −1.88627, −2.01959, −2.020631, −1.9998, −2.116455, −1.888353, −2.024797, −2.13312, −1.775864, −1.726911, −1.706079, −1.925849, −1.944597, −1.561302, −1.405068, −1.536305, −1.731077, −1.86648, −1.51339, −1.340491, −1.472769, −1.405068, −1.629004, −1.46652, −1.281122, −1.495684, −1.308203, −1.200922, −1.155093, −1.137386, −1.092599, −1.106139, −0.89991, −0.9926091, −1.049895, −0.5905659, −0.8384578, −0.3385078, −0.6676416, −0.8853281, −0.3822534, −0.5916075, 0.03749625, −0.5145319, −0.79992, −0.283305, −0.02187281, 0.4312069, −0.2697647, −0.5530697, −0.1656084, 0.3624637, 0.6468103, −0.06561844, 0.1041562, 0.1749825, 0.5134903, 0.7686731, 0.3885028, 0.7009715, 0.43329, 0.6759741, 0.7343016, 0.8519982, 0.8822035, 0.8717878, 0.9384478, 0.8957437, 1.060311, 1.101973, 1.263415, 1.116555, 1.103015, 1.359239, 1.468603, 1.520681, 1.308203, 1.156134, 1.560261, 1.632128, 1.518598, 1.23321, 1.16655, 1.641502, 1.622754, 1.530055, 1.538388, 1.359239, 1.536305, 1.571718, 1.467562, 1.58005, 1.404026, 1.651918, 1.658167, 1.441522, 1.489434, 1.344657<br>Phase C: −1.531097, −1.337366, −1.583175, −1.796695, −1.872729, −1.543596, −1.714412, −1.727952, −1.9196, −1.940431, −1.777947, −1.993551, −1.930015, −1.934182, −1.908142, −1.932098, −2.102915, −1.881062, −1.98001, −1.875854, −1.909184, −2.047712, −1.88627, −1.950847, −2.030005, −1.845649, −1.772739, −1.769615, −1.965428, −1.965428, −1.660251, −1.661292, −1.742534, −1.871688, −2.040421, −1.644627, −1.604006, −1.773781, −1.543596, −1.847732, −1.642544, −1.716495, −1.737326, −1.557136, −1.714412, −1.614422, −1.610256, −1.48006, −1.525889, −1.514432, −1.521723, −1.563385, −1.205088, −1.365488, −1.116555, −1.383195, −1.530055, −1.094682, −1.327992, −0.8176265, −1.210296, −1.462354, −1.092599, −1.01969, −0.4676616, −1.029064, −1.389444, −0.9738609, −0.5728593, −0.3228844, −0.9686531, −0.8853281, −0.6968053, −0.3905859, −0.1489434, −0.5582775, −0.4312069, −0.507241, −0.2551828, −0.1354031, −0.1812319, −0.06770156, −0.09582375, −0.03437156, −0.02603906, 0.04582875, −0.05311969, 0.1145719, 0.04687031, 0.0416625, 0.3791288, 0.3010116, 0.4457887, 0.2395594, 0.06666, 0.8457487, 0.8374162, 0.6780572, 0.3905859, 0.2093541, 0.8790787, 1.209254, 0.8874112, 0.9686531, 0.5311969, 1.010316, 1.304036, 0.9717778, 1.148843, 0.8301253, 1.200922, 1.400902, 1.167592, 1.372779, 1.189464, 1.275914, 1.464437, 1.412359, 1.443606, 1.359239, 1.531097, 1.559219, 1.465478, 1.53318, 1.385278, 1.618588, 1.664417, 1.465478, 1.635253, 1.310286, 1.582133, 1.650877, 1.427982, 1.321743, 1.163425, 1.636295, 1.604006, 1.426941, 1.214462, 1.181132, 1.500892, 1.4134, 1.443606, 1.208212, 1.194672, 1.320701, 1.190506, 1.112389, 1.0801, 1.041563, 0.9707363, 1.043646, 0.8519982, 0.9124088, 0.9384478, 0.7051378, 0.9061594, 0.5686931, 0.7842966, 0.9082425, 0.2551828, 0.08228344, 0.1447772, 0.5468203, 0.7634653, −0.1864397, −0.5103656, −0.1604006, 0.03228844, 0.4812019, −0.3124687, −0.6416025, −0.2135203, −0.4593291, 0.1760241, −0.4614122, −0.7520081, −0.3030947, −0.73326, −0.349965, −0.716595, −0.9196997, −0.7582575, −0.8894944, −0.9457387, −0.9676116, −1.123846, −1.176966, −1.13322, −1.34674, −1.224877, −1.261332, −1.426941, −1.390486, −1.554011, −1.362364, −1.203005, −1.520681, −1.597757, −1.800862, −1.481102, −1.343616, −1.547762, −1.793571, −1.874812, −1.539429, −1.705038, −1.702955, −1.863355, −1.959179, −1.811277, −2.06646, −1.820651, −1.978969, −1.809194, −1.934182, −2.076876, −1.897727, −2.065418, −1.806069, −1.917517, −2.092499, −1.928974, −1.998758, −2.056044, −1.785238, −1.782113, −1.736285, −1.982093, −1.969595, −1.65296, −1.608173, −1.759199, −1.907101, −1.934182, −1.654001, −1.64671, −1.797737, −1.564427, −1.850857, −1.600882, −1.620671, −1.822734, −1.660251, −1.715453, −1.618588, −1.631087, −1.507141, −1.469645, −1.451938
SA4	Phase A: 1.357156, 1.548803, 1.796695, 1.91335, 1.711287, 1.630045, 1.668583, 1.845649, 1.914392, 1.922724, 2.042504, 1.787321, 1.926891, 1.626921, 1.881062, 2.053961, 1.853981, 1.882103, 1.660251, 1.853981, 1.93314, 1.821693, 1.888353, 1.887311, 1.811277, 1.726911, 1.69983, 1.802945, 1.912309, 1.49985, 1.431107, 1.547762, 1.728994, 1.73316, 1.512349, 1.309244, 1.509224, 1.337366, 1.538388, 1.347782, 1.258208, 1.43319, 1.21967,

1.375904, 1.229044, 1.169675, 1.100932, 1.061352, 0.8155434, 0.9353231, 1.037396, 0.6947222, 0.8030447, 0.4228744, 0.7717978, 0.9686531, 0.2426841, 0.5697347, −0.0249975, 0.5009916, 0.7801303, 0.16665, −0.1291537, −0.3926691, 0.3208013, 0.6020231, 0.1301953, −0.4384978, −0.5884828, −0.05103656, 0.2947622, −0.1145719, −0.5218228, −0.6093141, −0.0083325, 0.03541312, −0.2114372, −0.6634753, −0.642644, −0.016665, −0.04791187, −0.3103856, −0.63327, −0.7530497, −0.01145719, −0.05416125, −0.3905859, −0.7197197, −0.7645069, −0.09686531, −0.13332, −0.3728794, −0.6468103, −0.7405509, −0.3405909, −0.1093641, −0.4145419, −0.6405609, −0.7728394, −0.416625, −0.2124788, −0.4937006, −1.038438, −0.9530297, −0.5041162, −0.3051778, −0.4978669, −1.224877, −1.123846, −0.6218128, −0.3822534, −0.6759741, −1.281122, −1.305078, −0.6697247, −0.3530897, −0.7717978, −1.381112, −1.294662, −0.6478519, −0.3822534, −0.5936906, −1.239459, −1.138428, −0.6551428, −0.3885028, −0.5405709, −1.085308, −0.9696947, −0.4780772, −0.2864297, −0.5364047, −0.7624238, −0.7551328, −0.2708063, −0.006249375, −0.3478819, −0.7322184, −0.7624238, −0.13332, −0.06666, −0.3957937, −0.7061794, −0.7915875, −0.01562344, −0.06666, −0.3978769, −0.6009815, −0.7311769, −0.06666, −0.03853781, −0.2676816, −0.6280622, −0.6593091, −0.01770656, 0.06874312, −0.26664, −0.5978569, −0.5405709, −0.09894843, 0.2853881, −0.09999, −0.4687031, −0.5259891, 0.2249775, 0.5780672, 0.01770656, −0.1978969, −0.3812119, 0.3124687, 0.6593091, 0.1853981, 0.4270406, 0.05520281, 0.5218228, 0.7717978, 0.4041263, 0.6874313, 0.4926591, 0.7520081, 0.9321985, 0.86658, 0.8676215, 0.9124088, 0.9957337, 1.055103, 1.264457, 1.071768, 1.342574, 1.148843, 1.151968, 1.439439, 1.282163, 1.557136, 1.377987, 1.215503, 1.498808, 1.690456, 1.7467, 1.457146, 1.409234, 1.55297, 1.781072, 1.837316, 1.6665, 1.642544, 1.734202, 1.824818, 1.817527, 1.894602, 2.056044, 1.761282, 1.868563, 1.64671, 1.898768, 2.027922, 1.827942, 1.915433, 1.622754, 1.839399, 1.921683, 1.838358, 1.915433, 1.871688, 1.798778, 1.689414, 1.678999, 1.836275, 1.915433, 1.467562, 1.401943, 1.594632, 1.667542, 1.839399, 1.400902, 1.331117, 1.515473, 1.341532, 1.600882, 1.360281, 1.220711, 1.51339, 1.262374, 1.39986, 1.257166, 1.257166, 1.003025, 1.065518, 0.8082525, 0.9644869

Phase B: −1.867522, −1.623796, −1.373821, −1.387361, −1.555053, −1.600882, −1.594632, −1.269665, −1.368613, −1.39986, −1.257166, −1.242584, −1.187381, −0.9874012, −1.109264, −1.118638, −0.9551128, −0.9644869, −0.8936606, −0.7884628, −0.8009616, −0.5811919, −0.7488834, −0.7717978, −0.4010016, −0.2374762, −0.29997, −0.6009815, −0.7020131, 0.2041463, 0.3416325, 0.05728594, −0.3989184, −0.4978669, 0.2510166, 0.6447272, 0.1937306, 0.3093441, −0.09061594, 0.4509965, 0.7645069, 0.2395594, 0.6093141, 0.2176866, 0.7051378, 0.9155334, 0.7926291, 0.8311669, 0.8113772, 0.9061594, 1.039479, 1.012399, 1.01344, 1.361322, 1.196755, 1.142594, 1.418608, 1.325909, 1.663375, 1.372779, 1.230085, 1.483185, 1.661292, 1.836275, 1.449855, 1.285288, 1.536305, 1.772739, 1.870646, 1.569635, 1.481102, 1.616505, 1.838358, 1.973761, 1.612339, 1.611297, 1.725869, 1.834192, 1.667542, 1.591508, 1.601923, 1.542554, 1.744617, 1.63317, 1.385278, 1.525889, 1.435273, 1.716495, 1.744617, 1.257166, 1.094682, 1.220711, 1.64671, 1.648793, 0.9582375, 0.6957638, 0.9728194, 1.409234, 1.637336, 0.79992, 0.4634953, 0.8447072, 1.039479, 1.383195, 0.6759741, 0.3510066, 0.8134603, 0.8707463, 1.223836, 0.5863997, 0.2749725, 0.4791188, 0.7082625, 0.7415925, 0.3864197, 0.1676916, 0.2676816, 0.63327, 0.4447472, 0.23331, 0.1208213, −0.3187181, 0.3645469, −0.02083125, −0.02708063, −0.06041063, −0.3687131, −0.03645469, −0.4155834, −0.2541412, −0.1458188, −0.4437056, −0.6749325, −0.7645069, −0.6405609, −0.6124387, −0.7134703, −0.8134603, −0.8322085, −0.9644869, −1.145719, −0.9894844, −0.9488634, −0.9571959, −1.292579, −1.510266, −1.175924, −1.130095, −0.96657, −1.492559, −1.556094, −1.36653, −1.212379, −1.234252, −1.641502, −1.748783, −1.545679, −1.337366, −1.338408, −1.631087, −1.836275, −1.697747, −1.389444, −1.388403, −1.748783, −1.953971, −1.710246, −1.569635, −1.488393, −1.815443, −2.023756, −1.687331, −1.830025, −1.636295, −1.931057, −2.073751, −1.89981, −1.978969, −1.607131, −1.963345, −2.102915, −1.890436, −1.965428, −1.670666, −1.981052, −2.121663, −1.912309, −1.963345, −1.775864, −2.047712, −2.190406, −1.784197, −1.858148, −1.555053, −1.882103, −2.018548, −1.778989, −1.470686, −1.337366, −1.837316, −1.914392, −1.64671, −1.335283, −1.321743, −1.589424, −1.654001, −1.48006, −1.28633, −1.265498, −1.376946, −1.273831, −1.190506, −1.111347, −0.9978169, −1.116555, −1.156134, −0.9030347, −0.9436556, −0.823876, −0.7384678, −0.7509665, −0.5645269, −0.7561744, −0.5582775, −0.4374563, −0.2020631, −0.3103856, −0.6655585, −0.4957837, 0.1853981, 0.3749625, −0.06457687, −0.3582975, −0.3603806, 0.2614322, 0.6561844, 0.1728994, 0.3780872, 0.03020531, 0.5093241, 0.7405509, 0.2562244, 0.5489035, 0.4041263, 0.6488934, 0.8228344, 0.749925, 0.8717878, 0.8228344, 0.9769856

Phase C: 0.4572459, 0.05416125, −0.4301653, −0.5457788, −0.1583175, −0.049995, −0.2416425, −0.5697347, −0.6061894, −0.5436956, −0.8363747, −0.592649, −0.8415825, −0.7103456, −0.8301253, −1.043646, −0.9374062, −0.9999, −0.8030447, −1.172799, −1.191548, −1.312369, −1.189464, −1.132178, −1.436315, −1.540471, −1.420691, −1.279039, −1.201963, −1.777947, −1.807111, −1.614422, −1.414442, −1.3332, −1.735243, −1.975844, −1.667542, −1.777947, −1.404026, −1.868563, −2.011257, −1.743576, −1.927932, −1.627962, −1.951888, −2.08625, −1.909184,

	−1.955013, −1.706079, −2.059169, −2.123746, −1.849815, −1.906059, −1.793571, −2.020631, −2.17999, −1.798778, −1.91335, −1.582133, −1.906059, −2.116455, −1.688373, −1.56651, −1.459229, −1.931057, −2.005008, −1.717537, −1.343616, −1.321743, −1.61338, −1.802945, −1.622754, −1.35299, −1.329034, −1.655043, −1.685248, −1.51339, −1.293621, −1.036355, −1.500892, −1.647752, −1.325909, −1.23321, −0.9696947, −1.464437, −1.51339, −1.13322, −1.126971, −0.9926091, −1.135303, −1.103015, −0.8801203, −0.9978169, −0.9144919, −0.6957638, −0.6113972, −0.6853482, −0.8592891, −0.8290837, −0.449955, −0.2489334, −0.4593291, −0.001041563, −0.4645369, −0.2135203, −0.1260291, −0.4010016, 0.2520581, −0.1145719, −0.04270406, −0.03333, 0.06770156, 0.5624437, 0.4228744, 0.249975, 0.09061594, 0.4228744, 0.723886, 0.7582575, 0.3905859, 0.2051878, 0.7572159, 0.89991, 1.165508, 0.5801503, 0.2853881, 0.8270006, 1.085308, 1.356114, 0.6051478, 0.3343416, 0.8290837, 1.361322, 1.510266, 0.9009516, 0.6520181, 0.9988585, 1.510266, 1.567552, 1.118638, 1.089474, 1.315493, 1.534222, 1.715453, 1.21342, 1.404026, 1.39986, 1.677957, 1.565468, 1.51339, 1.576926, 1.592549, 1.796695, 1.867522, 1.559219, 1.574842, 1.703996, 1.860231, 1.918558, 1.655043, 1.497767, 1.649835, 1.81336, 1.905018, 1.515473, 1.345699, 1.557136, 1.622754, 1.822734, 1.440481, 1.305078, 1.557136, 1.308203, 1.59984, 1.398818, 1.238418, 1.362364, 1.176966, 1.115513, 1.104056, 1.125929, 1.044687, 0.940531, 0.7447172, 0.8874112, 1.064477, 0.6655585, 0.8572059, 0.4572459, 0.7853381, 0.8801203, 0.3135103, 0.4728694, 0.00416625, 0.5020331, 0.7603406, 0.1853981, −0.2708063, −0.383295, 0.3291337, 0.4103756, 0.04895344, −0.4270406, −0.4530797, −0.1479019, −0.06145219, −0.216645, −0.583275, −0.5582775, −0.5603606, −0.8530397, −0.5791088, −0.7790887, −0.7145119, −0.8197097, −1.026981, −0.9374062, −1.047812, −0.816585, −1.075934, −1.176966, −1.283205, −1.190506, −1.222794, −1.407151, −1.550887, −1.523806, −1.272789, −1.316535, −1.669625, −1.817527, −1.617547, −1.449855, −1.337366, −1.791487, −2.034172, −1.728994, −1.778989, −1.560261, −1.909184, −2.058127, −1.748783, −1.895644, −1.798778, −1.971678, −2.097707, −1.882103, −1.965428, −1.668583, −2.036255
